# Analysis of a case of immune-related pancreatitis induced by anti-PD-1 antibody therapy for gastric cancer: A case report

**DOI:** 10.1097/MD.0000000000050285

**Published:** 2026-10-09

**Authors:** Fengyun Zhang, Wenhua Xiao

**Affiliations:** a Department of Internal Medicine, North China Electric Power University Hospital, Beijing, China; b Department of Oncology, The Fourth Medical Center of the PLA General Hospital, Beijing, China.

**Keywords:** adverse drug reaction, case report, gastric cancer, immune checkpoint inhibitor, immune-related pancreatitis, nivolumab

## Abstract

**Background:**

Immune-related pancreatitis is a rare but clinically significant adverse event associated with immune checkpoint inhibitor therapy. Early recognition and appropriate management are essential to prevent potentially serious outcomes. Herein, we report a case of suspected immune-related pancreatitis during nivolumab-based immunotherapy for metastatic gastric cancer.

**Case presentation:**

An 82-year-old female patient, post-gastrectomy for gastric cancer with subsequent cerebral metastases, multiple leptomeningeal metastases, subcutaneous metastases, and retroperitoneal lymph node metastases, received “nivolumab + apatinib” therapy for 4 cycles. On the second day of the fourth cycle of treatment, the patient developed mild epigastric pain, acid regurgitation, and elevated pancreatic enzyme levels, suggestive of immune-related pancreatitis. Alternative etiologies, including biliary disease, alcohol use, and hypertriglyceridemia, were excluded. The patient was classified as Grade 1 according to the Common Terminology Criteria for Adverse Events v5.0 and was managed with fasting, parenteral nutrition, octreotide, enzyme inhibition, and electrolyte balance adjustment. Pancreatic enzyme levels normalized, and symptoms resolved. Nivolumab and apatinib were subsequently withheld.

**Conclusion:**

This case highlights that immune-related pancreatitis may present with only mild abdominal symptoms despite marked pancreatic enzyme elevation during immune checkpoint inhibitor therapy. Clinicians should maintain a high index of suspicion for pancreatic immune-related adverse events, even in the absence of typical imaging findings, and systematically exclude alternative causes to ensure timely and appropriate management.

## 1. Introduction

Gastric cancer ranks fourth among all cancers worldwide, with approximately 42% of gastric cancer patients in China. The overall prognosis for gastric cancer is poor, as early detection rates are low, with 90% of cases diagnosed at advanced stages. Presently, the primary comprehensive treatments for gastric cancer involve surgery, chemotherapy, radiation therapy, or a combination of targeted therapies; yet, recurrence and metastasis rates remain high. In 2019, the US Food and Drug Administration approved the programmed cell death protein 1 (PD-1) inhibitor pembrolizumab (Keytruda) for the treatment of gastric or gastroesophageal junction adenocarcinoma. With the increasing application of immunotherapy, it has become imperative to pay due attention to the related adverse reactions it may bring. Immune-related pancreatitis is an uncommon but potentially serious immune-related adverse event (irAE) associated with immune checkpoint inhibitor (ICI) therapy, with a reported overall incidence of approximately 0.9% to 2.2% for clinically significant pancreatitis.^[1,2]^ The diagnosis of immune-related pancreatitis can be challenging because clinical symptoms are often mild or absent, and pancreatic enzyme elevation may be the only initial manifestation. Moreover, distinguishing immune-related pancreatitis from other causes of pancreatic injury during ICI therapy requires careful evaluation. Herein, a case report is presented of an elderly patient with gastric cancer who developed suspected immune-related pancreatitis following immunotherapy. This case is noteworthy because of the patient’s advanced age, atypical presentation with mild symptoms despite significant enzyme elevation, and successful conservative management without corticosteroid therapy.

## 2. Clinical data

An 82-year-old female patient presented to our department in March 2020 with recurrent gastric cancer, 3 months following a surgery performed 7 months earlier. In early 2018, the patient reported upper abdominal discomfort, which significantly worsened by October of the same year, leading to the discovery of a gastric mass during gastroscopy. A computed tomography (CT) scan of the upper abdomen revealed thickening of the gastric wall in the antrum. On November 28, 2018, laparoscopic radical surgery was conducted in our hospital’s general surgery department under general anesthesia to address distal gastric cancer. Postoperatively, the pathology report indicated ulcerative adenocarcinoma of the gastric cardia (moderately to poorly differentiated), with some components showing mucinous adenocarcinoma. The tumor measured 10.0 × 6.5 × 3.2 cm, infiltrating the entire thickness of the gastric wall and forming tumor nodules that extended to the outer fat tissue, with vascular invasion evident. There were no tumors found in the margins of the upper (gastric body) and lower (duodenum) cuts or in the omentum. Lymph node metastases were detected in the greater and lesser curvatures of the stomach (3/35, 3/20). The tumor was staged as pT4aN2. No adjuvant chemotherapy was administered postoperatively. By the end of March 2019, the patient experienced a decline in expressive abilities and limb weakness. A local head CT revealed multiple intracranial lesions suspected to be metastases, prompting the referral to our facility. An enhanced magnetic resonance imaging scan of the head conducted on April 11, 2019, confirmed multiple brain metastases, with significant enhancement of the meninges, indicating probable metastasis. Biochemical tests on April 11, 2019, revealed amylase levels of 42 U/L, lipase levels of 34 U/L, markedly elevated high-sensitivity C-reactive protein levels of 45.1 mg/L, decreased magnesium levels of 0.71 mmol/L, and potassium levels of 3.66 mmol/L. Urinary amylase was measured at 71 U/L. A full-body positron emission tomography–CT scan on April 12, 2019, indicated a suspicious lesion adjacent to the splenic flexure of the transverse colon following gastric cancer surgery, potentially indicative of metastasis. The scan also identified multiple lymph node metastases in the abdominal cavity, a possible subcutaneous metastatic lesion in the left back (posterior to the sixth rib on the left side), and multiple brain metastases with extensive surrounding low-density edema. Immunohistochemistry revealed the following profile: MLH-1 (−), MSH-2 (+), MSH-6 (+), PMS-2 (−), HER-2 (0), EBER (−). The preliminary diagnosis consisted of postoperative recurrence of moderately to poorly differentiated adenocarcinoma of the stomach (pT4aN2, stage IIIB), marked by deoxyribonucleic acid mismatch repair deficiency, multiple brain metastases, lymph node metastases in the abdominal cavity, and a subcutaneous metastatic lesion in the left back. On April 16, 2019, Gamma Knife therapy was administered to treat the brain metastases, leading to noticeable improvements in limb strength and appetite. Subsequently, the patient underwent 3 cycles of treatment on April 24, May 15, and June 12, 2019, involving the regimen: Nal-IRI 200 mg on day 1 + apatinib 250 mg daily (one cycle every 21 days). After the second cycle, the patient underwent a radiological efficacy assessment, showing a partial response (Fig. 1A–D). During the fourth treatment cycle on July 4, 2019, the patient reported mild pain below the xiphoid process, without lower back radiation or rebound tenderness. Laboratory findings showed elevated blood amylase levels of 242 U/L (normal range, 0–110 U/L), lipase levels of 564 U/L (normal range, 0–60 U/L), and urinary amylase levels of 921 U/L. Abdominal ultrasound revealed the following: slight dilation of the extrahepatic bile duct in the upper segment; a small amount of sedimentation observed in the gallbladder cavity; and normal size of the pancreas with clear boundaries and mild dilation of the main pancreatic duct. The patient had no history of alcohol consumption, cholecystitis, cholelithiasis, or hyperlipidemia. Serum triglyceride and calcium levels were within normal limits. Positron emission tomography–CT performed prior to immunotherapy showed no evidence of pancreatic metastasis. Apatinib, a small-molecule tyrosine kinase inhibitor targeting vascular endothelial growth factor receptor 2, has rarely been reported to cause pancreatitis; furthermore, the patient had been receiving apatinib since the first cycle without pancreatic enzyme abnormalities, making apatinib an unlikely causative agent. After systematically excluding biliary obstruction, metabolic, infectious, and drug-related etiologies, and considering the temporal association between the onset of symptoms and nivolumab administration, immune-related pancreatitis was considered the most likely diagnosis. According to the Common Terminology Criteria for Adverse Events v5.0 grading criteria, the patient’s presentation was consistent with Grade 1 pancreatitis (enzyme elevation with mild symptoms, not requiring urgent intervention).^[3]^ Although abdominal ultrasound showed no pancreatic edema or enlargement, it should be noted that ultrasound has limited sensitivity for detecting mild pancreatitis; contrast-enhanced CT or magnetic resonance imaging was not performed given the mild clinical presentation and the patient’s advanced age. Immediate symptomatic treatment was provided with fasting, intravenous fluids, and octreotide, resulting in normalization of most biochemical parameters (Fig. 2A–C) and significant improvement in symptoms such as upper abdominal pain. Corticosteroid therapy was not administered because, according to current American Society of Clinical Oncology (ASCO) and National Comprehensive Cancer Network guidelines, Grade 1 immune-related pancreatitis can be managed with close monitoring and symptomatic treatment without the need for corticosteroids.^[4]^ Following recovery, both nivolumab and apatinib were withheld. During subsequent follow-up, pancreatic enzyme levels returned to normal, and no recurrence of pancreatitis or evidence of long-term endocrine or exocrine pancreatic insufficiency was observed. The timeline of the patient’s entire clinical course is summarized in Figure 3.

**Figure 1.**
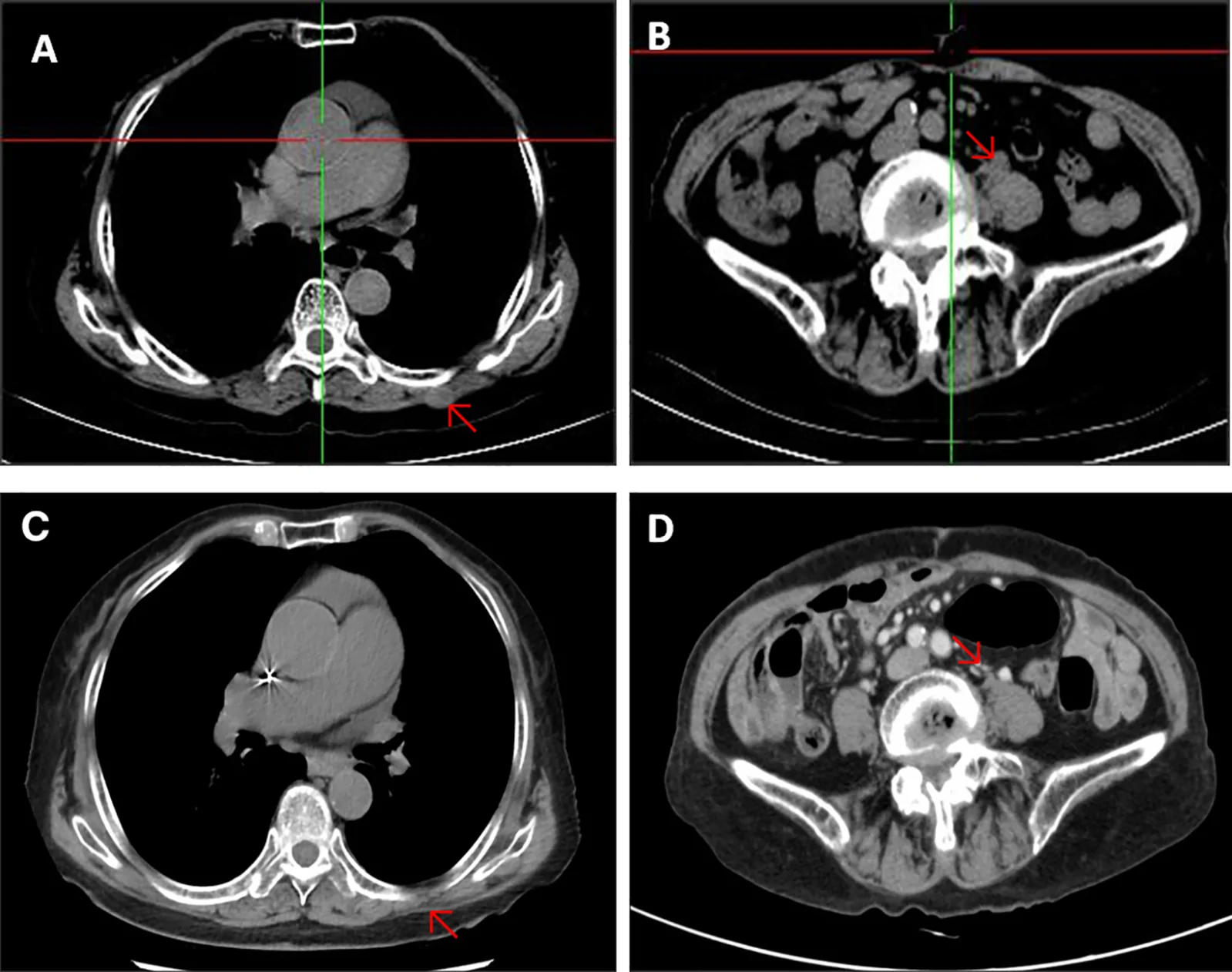
CT reexamination of the chest, abdomen, and pelvis indicated partial remission of the tumor. (A) Baseline assessment before treatment on April 12, 2019: subcutaneous metastatic tumor on the left back (left posterior sixth rib); the lesion demonstrated increased FDG uptake on PET-CT. (B) Baseline assessment before treatment on April 12, 2019: multiple abdominal lymph node shadows, SUVmax 4.21, suggestive of metastasis. (C, D) Assessment on June 11, 2019, and June 12, 2019: the subcutaneous metastasis of the left back disappeared, and the intraperitoneal lymph nodes shrank, indicating a partial response to immunotherapy. CT = computed tomography, PET-CT = positron emission tomography–computed tomography.

**Figure 2.**
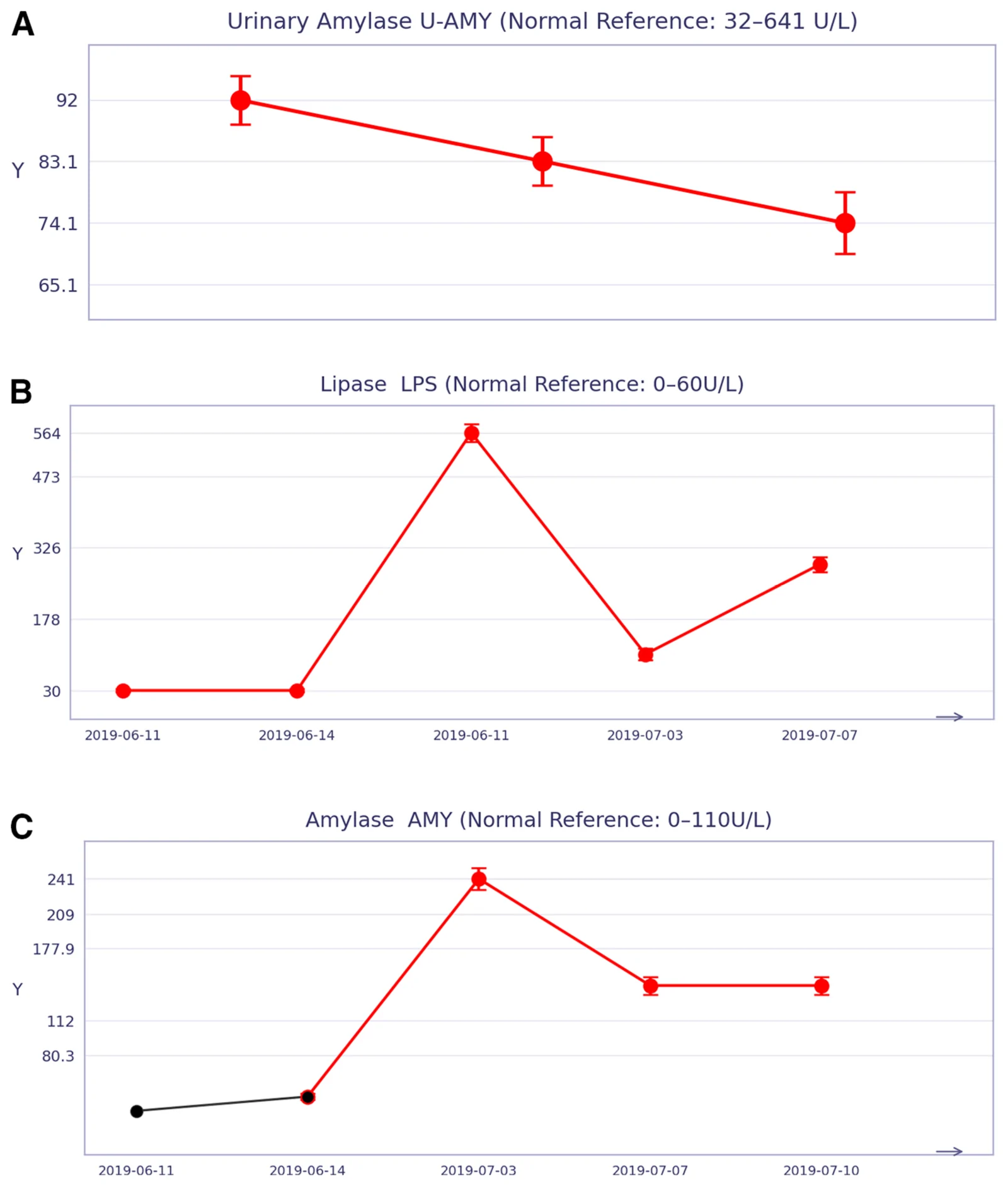
Trend of changes in various biochemical indicators during and after the episode of immune-related pancreatitis. (A) Urine amylase (U-AMY) trend, showing an initial peak followed by gradual normalization; (B) lipase (LPS) trend, demonstrating marked elevation (>9 × ULN) at onset with subsequent decline to normal levels; (C) blood amylase (AMY) trend, showing a moderate elevation that resolved with conservative treatment. ULN = upper limit of normal.

**Figure 3.**
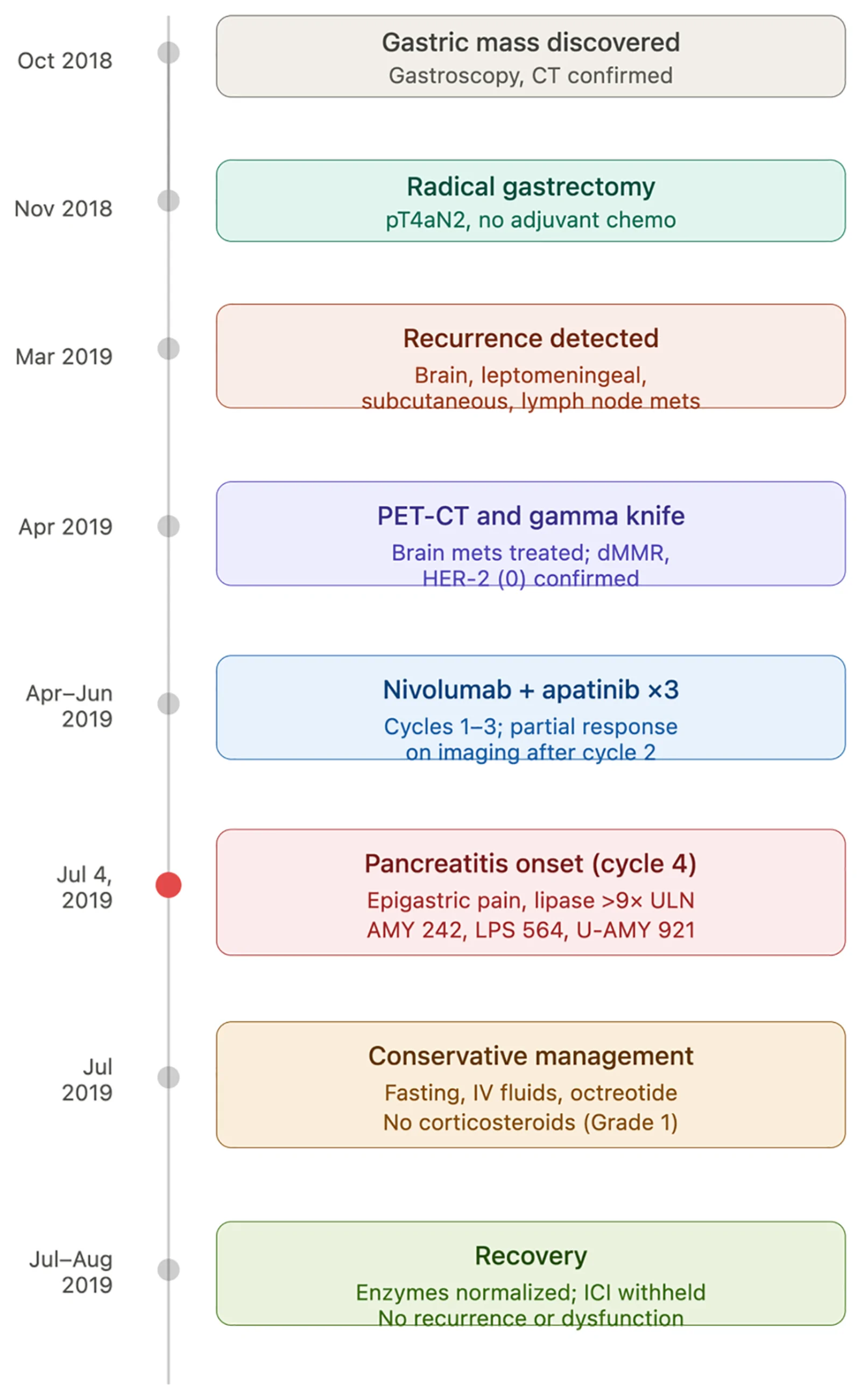
Timeline of the patient’s clinical course. A timeline figure illustrating the key events: initial gastric cancer diagnosis (2018) → radical gastrectomy (November 2018) → postoperative recurrence with brain metastases (March 2019) → gamma knife therapy (April 2019) → initiation of nivolumab + apatinib immunotherapy (April 2019) → onset of pancreatitis during the fourth cycle (July 2019) → conservative management and recovery → immunotherapy withheld. AMY = amylase, CT = computed tomography, ICI = immune checkpoint inhibitor, LPS = lipase, PET-CT = positron emission tomography–computed tomography, U-AMY = urine amylase, ULN = upper limit of normal.

## 3. Discussion

Currently, immunotherapy has been applied in various tumor types with promising therapeutic effects. For instance, pembrolizumab has demonstrated a 6-month survival rate of 80.2% in the treatment of non-small cell lung cancer. Immunotherapy, as an emerging approach in cancer treatment, has garnered significant attention, alongside the emergence of related adverse reactions, namely irAEs. Initially, pretreatment screening of favorable populations or specific groups is recommended. However, patients with autoimmune diseases, viral hepatitis, a history of hematopoietic stem cell or organ transplantation, as well as those with compromised general health, may potentially face ICI-related toxicities or other unforeseen toxic risks.

After completing 3 cycles of immunotherapy, the patient presented with abdominal pain and increased levels of amylase, lipase, and urinary amylase during the fourth cycle. After alternative etiologies were systematically excluded as described above, immune-related pancreatitis was considered the most likely diagnosis. The clinical presentation was classified as Grade 1 according to the Common Terminology Criteria for Adverse Events v5.0, and the patient’s symptoms were markedly alleviated by conservative treatment, including fasting, intravenous fluids, and octreotide, without the use of corticosteroids. Although the incidence of ICI-related pancreatitis is relatively low, ranging from 0.5% to 4%, it can significantly impair quality of life and even pose a life-threatening risk.^[5]^ A recent meta-analysis of 59 randomized controlled trials involving 41,757 patients reported an overall incidence of ICI-associated pancreatitis of 0.93%, with the incidences of amylase and lipase elevation being 2.57% and 2.78%, respectively.^[2]^ ICI-associated pancreatitis represents an extrinsic or intrinsic pancreatic toxicity within the spectrum of irAEs. Fang et al demonstrated that male gender and PD-1/programmed cell death ligand 1 blockade agents, particularly nivolumab, may serve as predisposing factors for immune-related pancreatitis.^[6]^

ICI-associated pancreatitis does not follow a specific periodic pattern. Literature reports suggest that immune-related pancreatitis often occurs between 2 and 16 weeks after the initiation of treatment.^[7,8]^ Some studies indicate that ICI-related pancreatitis may even manifest on the first day of ICI treatment.^[9]^ In some patients, symptoms appear over a year into treatment or after treatment cessation.^[10,11]^ Therefore, ICI-related pancreatitis can emerge at any point during the course of treatment. It is essential to consider the possibility of immune-related pancreatitis if any new pancreatic injury symptoms, such as abdominal pain or elevated pancreatic enzymes, arise. During the fourth cycle of immunotherapy, the patient exhibited an elevation in pancreatic enzymes, particularly a significant increase in lipase levels exceeding 9 times the upper limit of normal, alongside mild epigastric discomfort. No pancreatic edema or enlargement was observed on ultrasound, prompting initial consideration of mild pancreatitis. The patient had no history of cholecystitis or hyperlipidemia, and other triggering factors for pancreatitis were clinically ruled out. In the course of immunotherapy, a relatively high incidence of isolated pancreatic enzyme elevation has been noted, leading to primary consideration of enzyme elevation in association with the use of ICIs.

Compared with previously published cases of nivolumab-associated pancreatitis, the present case shares several common features, including a temporal association between ICI administration and symptom onset, elevated lipase as the predominant laboratory finding, and improvement after ICI discontinuation.^[6,12]^ However, this case is distinct in that the patient was elderly (82 years), presented with only mild abdominal symptoms despite markedly elevated lipase levels, and was successfully managed without corticosteroid therapy. In a previously reported case of nivolumab-induced autoimmune pancreatitis, the patient presented with cholestatic liver disease and required steroid and immunosuppressive therapy,^[12]^ whereas our patient’s presentation was considerably milder.

Studies indicate that for moderate pancreatitis (Grade 2), ICI treatment should be discontinued, and prednisone/methylprednisolone at a dosage of 0.5 to 1 mg/kg·d should be administered until symptoms improve to less than Grade 1. In cases of severe and life-threatening pancreatitis, it is recommended to permanently discontinue immunotherapy and initiate treatment with 1 to 2 mg/kg·d of glucocorticoids. Steroids can be gradually tapered over 4 to 6 weeks once symptoms of ICI-related pancreatitis improve to Grade 1.^[4]^ According to the updated ASCO guidelines, for Grade 1 pancreatitis, ICI therapy may be continued with close monitoring, and corticosteroid therapy is not required.^[3]^ In the present case, the decision to manage the patient conservatively without corticosteroids was based on these guideline recommendations. However, given the patient’s advanced age and overall clinical condition, both nivolumab and apatinib were withheld as a precautionary measure. Due to the lack of higher-level evidence, active observation is often employed for asymptomatic individuals with isolated enzyme elevation of pancreatic impairment, and there is no clear guidance on whether to discontinue PD-1 monoclonal antibodies. However, small-scale studies have shown that ICIs can also lead to fatal pancreatitis. In patients with isolated enzyme elevation, 14% of patients were found to have clinically significant pancreatitis during long-term follow-up,^[13]^ while others were observed to have pancreatic atrophy and decreased exocrine and endocrine function. It has been reported that PD-1 monoclonal antibody therapy can lead to isolated elevation of amylase and lipase in up to 14% and 8% of patients, respectively. In the absence of clinical symptoms and only with biochemical changes, there is no need for active treatment or discontinuation of the medication.

The understanding of ICIs’ impact on pancreatic damage in the early stages remains insufficient. There is a consensus among experts that routine testing for pancreatic enzymes is unnecessary. Instead, close monitoring of isolated elevation of pancreatic enzymes is recommended, without the need to discontinue immunotherapy or undertake active intervention. A retrospective analysis of 15 clinical trials involving 9099 patients revealed that single-agent immune therapy can lead to elevated pancreatic enzymes, with a higher incidence observed in combined immune therapy, indicating an association between immune therapy and pancreatic impairment of unclear significance. However, clinically significant pancreatitis induced by ICIs compared with chemotherapy or placebo shows a similar outcome. Nevertheless, case reports in recent years have highlighted fatal pancreatitis caused by ICIs. Long-term observations on small samples have shown that 13% of patients with isolated elevation of pancreatic enzymes progress to clinically significant pancreatitis, and some patients may experience insufficient pancreatic exocrine and endocrine function even after enzyme levels return to normal.

Therefore, the elevation of isolated pancreatic enzymes holds some instructive value, signaling the presence of pancreatic damage. It is crucial to explore more sensitive and specific indicators for early pancreatic injury detection in the future. It should be acknowledged that this is a single case report, and a definitive causal relationship between nivolumab and the pancreatic injury cannot be established despite the temporal association and the exclusion of other common etiologies. Finally, in cases of adverse reactions during or after treatment, it is advisable to refer to guidelines from the European Society for Medical Oncology, the National Comprehensive Cancer Network, ASCO, the Society for Immunotherapy of Cancer, and the Chinese Society of Clinical Oncology for diagnosing and grading immune-related adverse reactions, thereby ensuring appropriate management of relevant adverse events. In conclusion, this case illustrates that immune-related pancreatitis may present with only mild or atypical symptoms despite significant pancreatic enzyme elevation. Clinicians should maintain a high index of suspicion for pancreatic irAEs during ICI therapy, systematically exclude alternative causes, and manage patients according to established grading and treatment guidelines.

## Author contributions

**Conceptualization:** Fengyun Zhang.

**Data curation:** Wenhua Xiao.

**Formal analysis:** Wenhua Xiao.

**Investigation:** Fengyun Zhang, Wenhua Xiao.

**Project administration:** Wenhua Xiao.

**Software:** Wenhua Xiao.

**Supervision:** Fengyun Zhang.

**Writing – original draft:** Fengyun Zhang.

**Writing – review & editing:** Wenhua Xiao.
